# Histological Analysis of γδ T Lymphocytes Infiltrating Human Triple-Negative Breast Carcinomas

**DOI:** 10.3389/fimmu.2014.00632

**Published:** 2014-12-10

**Authors:** Jose Villacorta Hidalgo, Peter Bronsert, Marzenna Orlowska-Volk, Liliana B. Díaz, Elmar Stickeler, Martin Werner, Annette Schmitt-Graeff, Gian Kayser, Miroslav Malkovsky, Paul Fisch

**Affiliations:** ^1^Department of Pathology, University of Freiburg Medical Center, Freiburg im Breisgau, Germany; ^2^Faculty of Biology, University of Freiburg, Freiburg im Breisgau, Germany; ^3^University Hospital “José de San Martin”, University of Buenos Aires, Buenos Aires, Argentina; ^4^Comprehensive Cancer Center, Freiburg im Breisgau, Germany; ^5^Department of Obstetrics and Gynecology, University of Freiburg Medical Center, Freiburg im Breisgau, Germany; ^6^Department of Medical Microbiology and Immunology, University of Wisconsin School of Medicine and Public Health, Madison, WI, USA

**Keywords:** γδ T-cells, breast cancer, triple-negative breast cancer, histology, paraffin material

## Abstract

Breast cancer is the leading cause of cancer death in women and the second most common cancer worldwide after lung cancer. The remarkable heterogeneity of breast cancers influences numerous diagnostic, therapeutic, and prognostic factors. Triple-negative breast carcinomas (TNBCs) lack expression of HER2 and the estrogen and progesterone receptors and often contain lymphocytic infiltrates. Most of TNBCs are invasive ductal carcinomas (IDCs) with poor prognosis, whereas prognostically more favorable subtypes such as medullary breast carcinomas (MBCs) are somewhat less frequent. Infiltrating T-cells have been associated with an improved clinical outcome in TNBCs. The prognostic role of γδ T-cells within CD3^+^ tumor-infiltrating T lymphocytes remains unclear. We analyzed 26 TNBCs, 14 IDCs, and 12 MBCs, using immunohistochemistry for the quantity and patterns of γδ T-cell infiltrates within the tumor microenvironment. In both types of TNBCs, we found higher numbers of γδ T-cells in comparison with normal breast tissues and fibroadenomas. The numbers of infiltrating γδ T-cells were higher in MBCs than in IDCs. γδ T-cells in MBCs were frequently located in direct contact with tumor cells, within the tumor and at its invasive border. In contrast, most γδ T-cells in IDCs were found in clusters within the tumor stroma. These findings could be associated with the fact that the patient’s prognosis in MBCs is better than that in IDCs. Further studies to characterize these γδ T-cells at the molecular and functional level are in progress.

## Introduction

Worldwide, breast cancer is the principal cause of cancer related deaths in women in developed and in developing countries (1). Breast cancer is a heterogeneous disease of numerous tumor subtypes with different biological characteristics and clinical prognosis (2). One subgroup with a particularly poor prognosis are triple-negative breast carcinomas (TNBCs) characterized by lack of estrogen receptor (ER), progesterone receptor (PR), and human epidermal growth factor receptor 2 (HER2) expression. TNBCs account for 10–17% of all breast carcinomas, depending on the sensitivity of tests used to define the ER, PR, and HER2 status (3) and frequently, these tumors contain marked lymphocytic infiltrates (4). TNBCs are generally high-grade tumors and mostly invasive ductal carcinomas (IDCs), although other types of breast cancers can also be triple negative such as the medullary breast carcinoma (MBC) (5, 6). MBCs represent only 3–5% of all breast cancers and are characterized by a well-circumscribed margin, a poorly differentiated nuclear grade, a high-mitotic rate, prominent syncytial growth in more than the 75% of the tumor area and a diffuse lymphoid infiltrate without intraductal components or micro-glandular features (7). And although the MBC’s aggressive histological characteristics are very similar to those of high-grade triple-negative IDCs, MBCs have generally a remarkably better prognosis than IDCs (7–9).

The association between tumor-infiltrating lymphocytes (TILs) and the clinical outcome has been well established in many different cancers and these findings initiated an increasing interest in valid markers of tumor behavior and treatment response (10–13). However, the numbers and composition of TILs may vary depending on the types of immune responses and antigens (14). Prominent infiltration by CD8^+^ T-cells has been generally associated with a better prognosis and response to therapies (15–18). In contrast, a predominance of some CD4^+^ T-cell subsets within TILs has been linked to a poorer outcome while the prognostic significance of increased numbers of regulatory T-cells (Tregs) remain controversial and may depend on the type of tumor (19, 20).

In this context, γδ T-cells have been studied in distinct cancers as an interesting and intriguing part of the tumor microenvironment with demonstration of cytotoxicity *in vitro* against both solid and hematological malignancies (21–25). However, the identification and relevance of the different γδ T-cell subsets within the tumor microenvironment remain poorly characterized. Vγ9Vδ2 T lymphocytes are the main subset in the human adult peripheral blood, where γδ T-cells typically constitute about 5% of CD3^+^ lymphocytes. Besides Vγ9Vδ2 T-cells, lymphocytes expressing Vδ1 are typically found in human tissues (26, 27) such as intestine, mucosa, and skin. Even though they constitute only a small population of lymphocytes, γδ T-cells may play a non-overlapping role in some human infections, autoimmunity (28), and tumor microenvironment (29, 30). The Vγ9Vδ2 T-cell subset recognizes phosphoantigens such as isopentenyl pyrophosphate (IPP). IPP is produced in all higher eukaryotic cells including human cancer cells by the mevalonate pathway. In contrast, many bacteria such as *Mycobacterium tuberculosis* and protozoa such as *Malaria* parasites use the non-mevalonate (1-deoxy-d-xylulose-5-phosphate; DOXP) pathway for the phosphoantigenic biosynthesis. (31). These antigens are presented to human Vγ9Vδ2 T-cells bound to the intracellular B30.2 domain of butyrophilin 3A1 (32). Antigens recognized by other human γδ T-cell subsets remain poorly defined. It has been suggested that Vδ1 recognize MHC class I related molecules MICA, MICB, and ULBPs (21, 33). Infiltration by γδ T-cells in human breast carcinomas and a potential role of cytotoxic Vγ9δ2 T-cells against breast cancer cells has been initially described by Bank et al. in 1993 (34).

Here, we analyzed the presence of γδ T-cells in the human TIL immune microenvironment of 26 TNBCs comparing triple-negative IDC and triple-negative MBC specimens. Since the amounts of TILs in primary TNBCs appear to be associated with prognosis (35), we studied these tumors, focusing on the possibility that immunohistochemistry (IHC) of γδ T-cell infiltration may help our understanding of the substantial prognostic difference between IDCs and MBCs.

## Materials and Methods

### Tissue specimens

We analyzed 30 formalin-fixed, paraffin-embedded (FFPE) specimens from patients with *TNBCs* that were obtained between 2003 and 2011 and preserved in the archives of the Institute of Clinical Pathology of the Freiburg University Medical Center. From these, we selected 14 IDC and 12 MBC samples with an equivalent lymphocytic infiltration of at least 50% of the sample area in HE staining (Table 1). All specimens in this study were obtained before the patients were treated with chemotherapy or radiotherapy. In addition, we analyzed for comparison non-malignant breast tissues (11 normal breast tissues and 7 fibroadenomas). Controls included sections from two TCRγδ lymphomas (kindly provided by Prof. Müller-Hermelink, Würzburg). Negative controls included TCRαβ lymphomas and isotype controls. The age of the patients ranged between 43 and 82 years (median 57 years). Type of tumor and staging were performed according to the classification of the Union for International Cancer Control (UICC). All tumors included in this study were grade III according to the modified Bloom–Richardson classification (36). *MBC* was diagnosed using the Ridolfi criteria (7). The Ethics Committee of the University of Freiburg Medical Center approved the use of the patient materials in this study for morphologic analyses.

**Table 1:** **Characteristics of patients with grade 3 triple negative tumors**.

Diagnosis	Age (mean)	Tumor size mean (cm)	Stage (TNM)^a^		
			N0	N1	N2
IDC (*n* = 14)	57.5 (±11.7)	2.7 (±1.93)	10^b^	1	3
MBC (*n* = 12)	59.1 (±13.3)	2.2 (±1.28)	7	4	1

*^a^All patients were M0*.

*^b^Numbers of patients*.

### Immunohistochemistry

Sections (2 μm) were mounted on Superfrost plus^®^ Adhesion glass slides (R. Langenbrinck, Emmendingen, Germany. Code 03-0060) after dewaxing and rehydration. Antigen retrieval was performed using the buffers as detailed in Table 2. Endogenous peroxidase activity was blocked by the peroxidase blocking reagent (EnVision™ FLEX Systems FLEX, Dako, Carpinteria, CA, USA. Code S2023) for the rabbit CD3 antiserum that was detected by the peroxidase based detection system. For the alkaline phosphatase based detection method [anti-TCRγδ monoclonal antibody (mAb) and caspase-3 polyclonal antiserum], non-specific protein binding was blocked using 3% BSA in PBS. Antigen retrieval was performed in citrate buffer at pH 6 in a pressure cooker (anti-TCRγδ mAb), in Dako pH 6.1 buffer (anti-caspase-3 antiserum), or in Dako pH 9 EDTA buffer (anti-CD3 antiserum) in a steam cooking machine (Table 2). The use of a microwave oven did not produce good results. Sections were incubated with primary antibodies that were rabbit-anti-human CD3 polyclonal antiserum (EnVision™ FLEX Systems Dako. Code IS503, undiluted), mouse anti-human TCRγδ mAb (clone γ3.20, Thermo Scientific, Germany. Code 10772535, 1:40) and rabbit-anti-human cleaved-caspase-3 antiserum (Cell Signall Corp., Danvers, MA, USA, Code 9662S, 1:700). Horseradish peroxidase-conjugated secondary antibodies (EnVision™ FLEX Systems Dako, Code 5007) and alkaline phosphatase-conjugated secondary antibodies (Dako REAL™ Detection System, Alkaline Phosphatase/RED, rabbit/mouse, Code K5005) were employed for detection of the primary antibodies, a blue chromogen was used to detect the cleaved-caspase-3 antibody (Dako BCIP/NBT Substrate System Code K0598) and hematoxylin was used as a counterstain.

**Table 2:** **Antibodies used in immunohistochemistry**.

Antibody	Dilution	Retrieval buffer	Incubation time (min)
Anti-TCRγδ mAb	1:40	Citrate pH 6	30
CD3 antiserum	Undiluted	Dako pH 9	30
Caspase-3 c antiserum	1:700	Dako pH 6.1	45

### Microscopy

Immunohistochemistry was analyzed using an Axioplan^®^ microscope (Carl Zeiss, Jena, Germany), equipped with a Axiocam^®^ MRc (Carl Zeiss), digital camera. Twenty randomly selected high-power fields (HPF) of each sample were photographed (10 from the tumor parenchyma and 10 from the stroma). A HPF 400× was defined using a 40× objective and a 10× ocular magnification equipped with a 26-mm ocular reticule (Carl Zeiss). For the caspase-3 analysis, 20 HPF were considered from tumor areas. The cells were counted manually in all sections by two different investigators.

### Statistical analysis

The unpaired *t*-test using the GraphPad Prism Software (GraphPad Inc., San Diego, CA, USA. Version 6) was used for statistical analysis.

## Results

### Distribution of TCRγδ^+^ T-cells in normal breast tissues

**Figure 1:**
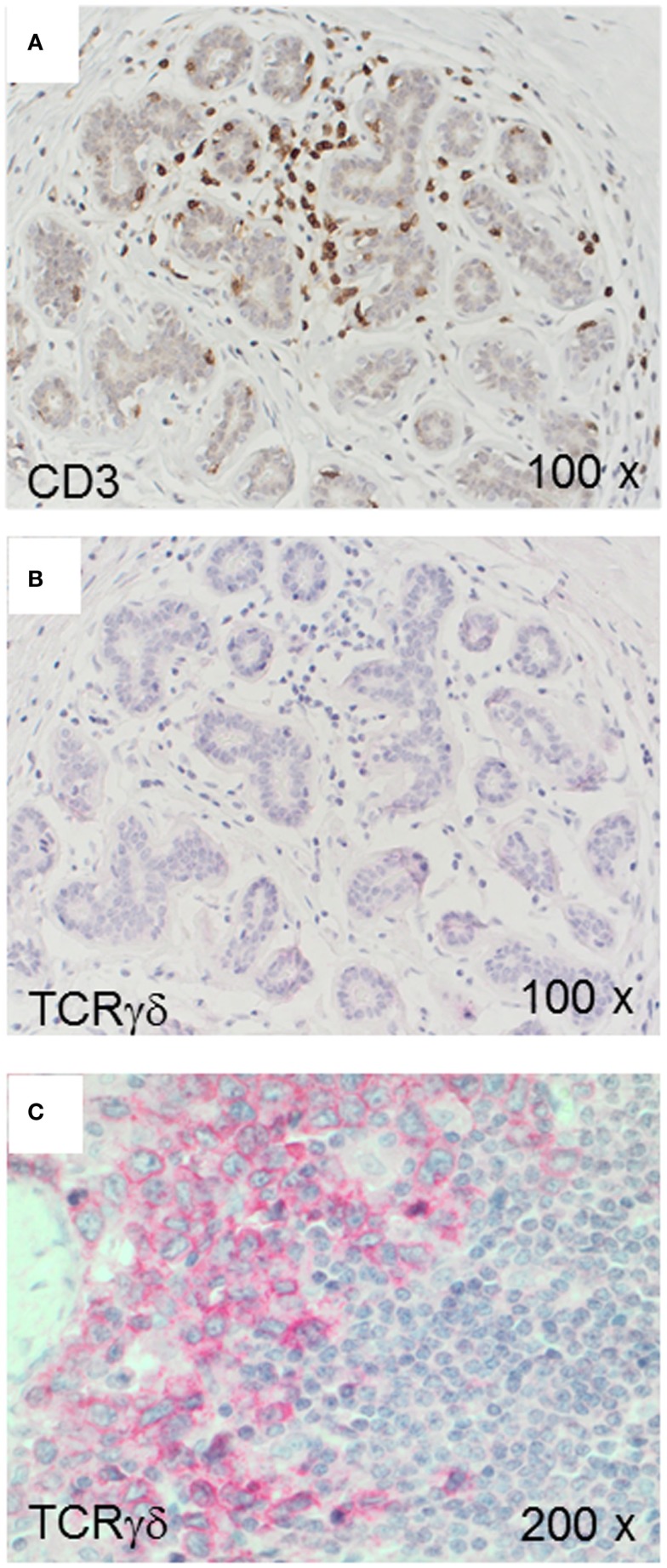
**CD3^+^ and TCRγδ^+^ T-cells in normal breast tissue**. Representative normal breast tissue stained for CD3^+^ **(A)** and TCRγδ^+^ **(B)** T-cells. Controls included a TCRγδ^+^ T-cell lymphoma involving the stomach **(C)**. CD3^+^ cells are detected by the brown chromogen **(A)** while TCRγδ^+^ cells are stained red **(B,C)**.

We used the mAb γ3.20 that is able to detect γδ T-cells in paraffin-embedded material (37) for IHC studies of normal breast tissues (*n* = 11). There were only few CD3^+^ cells in normal breast sections (Figure 1A) and very few if any expressed the TCRγδ (Figure 1B). In contrast, infiltrations by γδ T-cells in FFPE samples of TCRγδ lymphomas (Figure 1C) stained positive by the anti-TCRγδ mAb. γδ T-cells were also detectable in tonsils and other normal human tissues (data not shown).

### TCRγδ^+^ cells in IDCs and MBCs

**Figure 2:**
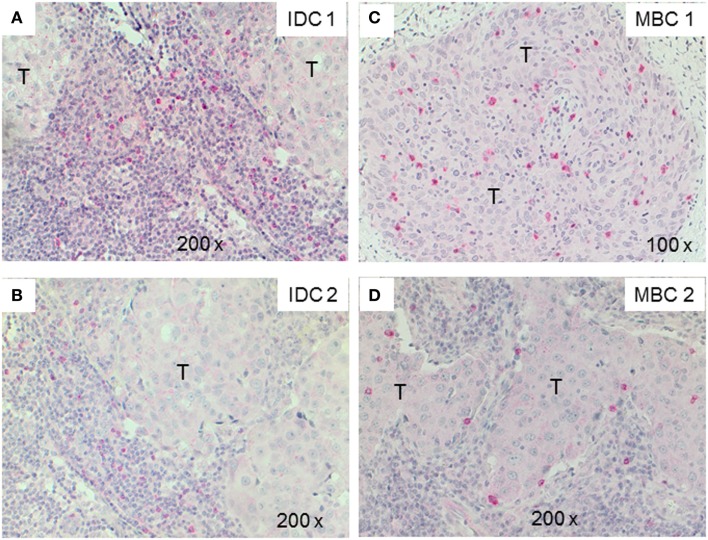
**TCRγδ^+^ T-cells in representative triple negative invasive ductal carcinomas (IDCs) and medullary breast carcinomas (MBCs)**. Two representative cases of IDC **(A,B)** and MBC **(C,D)** were stained by IHC for TCRγδ^+^ T-cells. The tumor area is marked with T, TCRγδ^+^ T-cells are detected by the red chromogen. The tumor area is marked (“T”).

**Figure 3:**
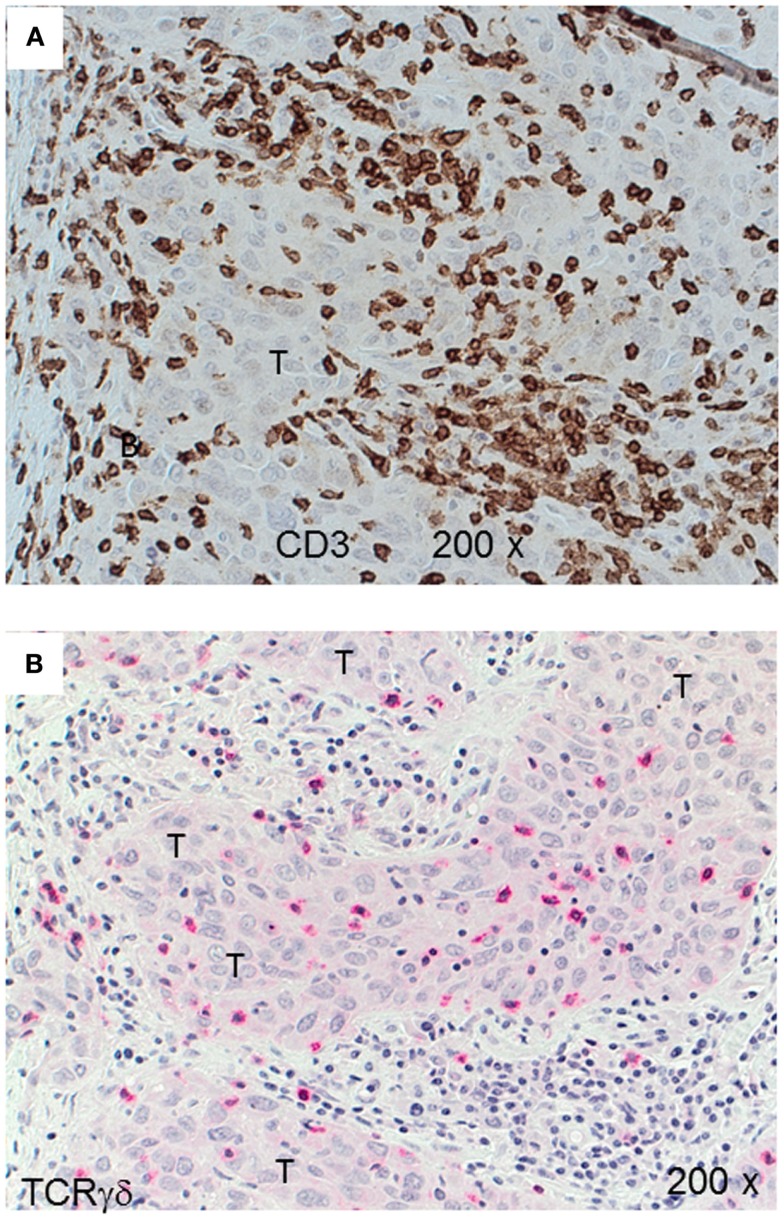
**CD3^+^ and TCRγδ^+^ T-cells in an invasive ductal carcinoma (IDC)**. IHC of an IDC with a rich lymphocytic infiltration that extends into the tumor parenchyma (T). CD3^+^ T-cells are detected by the brown chromogen **(A)** while TCRγδ^+^ cells are stained red **(B)**. The tumor area is marked (“T”).

Next, we examined triple-negative IDCs (*n* = 14) and MBCs (*n* = 12) for the presence of γδ T-cells since these tumors are frequently infiltrated by lymphocytes (38). Indeed, the lymphocytic infiltrates in IDCs and MBCs contained many γδ T-cells (Figure 2). Although both types of TNBCs, contained conspicuous numbers of γδ T-cells (Table 3), the TCRγδ^+^ cells within the TILs were more frequently located in the stroma of the IDC sections (Figures 2A,B), while in the MBC sections TCRγδ^+^ cells were typically located in the tumor parenchyma (Figure 2C) and at the invasive tumor cell border (Figure 2D). Nevertheless, this distinction was not absolute since individual IDC cases contained many γδ T-cells both in the tumor stroma and parenchyma (Figure 3). However, there were significantly more TCRγδ^+^ cells within the tumor parenchyma in MBCs than in IDCs (Table 3).

**Table 3:** **CD3^+^ and TCRγδ^+^ T-cells in the stroma and parenchyma of IDC and MBC**.

	CD3^+^ cells		TCRγδ^+^ cells		Cleaved-caspase-3^+^ cells
	Stroma	Parenchyma	Stroma	Parenchyma	Tumor
IDC (*n* = 14)	27 (±9)^a^	16 (±7)*	6 (±4)	2 (±2)**	4 (±2)*
MBC (*n* = 12)	24 (±2)	24 (±9)	4 (±2)	8 (±4)	7 (±3)

*^a^The numbers reflect the mean positive cells per HPF (determined from the means of a total of 20 HPF counted for each patient) of IDC (*n* = 14) and MBC patients (*n* = 12). SD reflects the standard variation within the cohorts of IDC and MBC patients*.

*The difference between the CD3^+^ cells within stroma and parenchyma was statistically significant in IDC (*p* < 0.05) but not in MBC (*p* = 0.901). In addition, apoptotic (CC3^+^) cells were significantly higher in MBC than in IDC (*p* < 0.05).

**The difference in the higher amount of TCRγδ T-cells in the tumor parenchyma in MBC than in IDC was statistically highly significant (*p* < 0.001).

### TCRγδ^+^ cells in fibrocystic breasts

**Figure 4:**
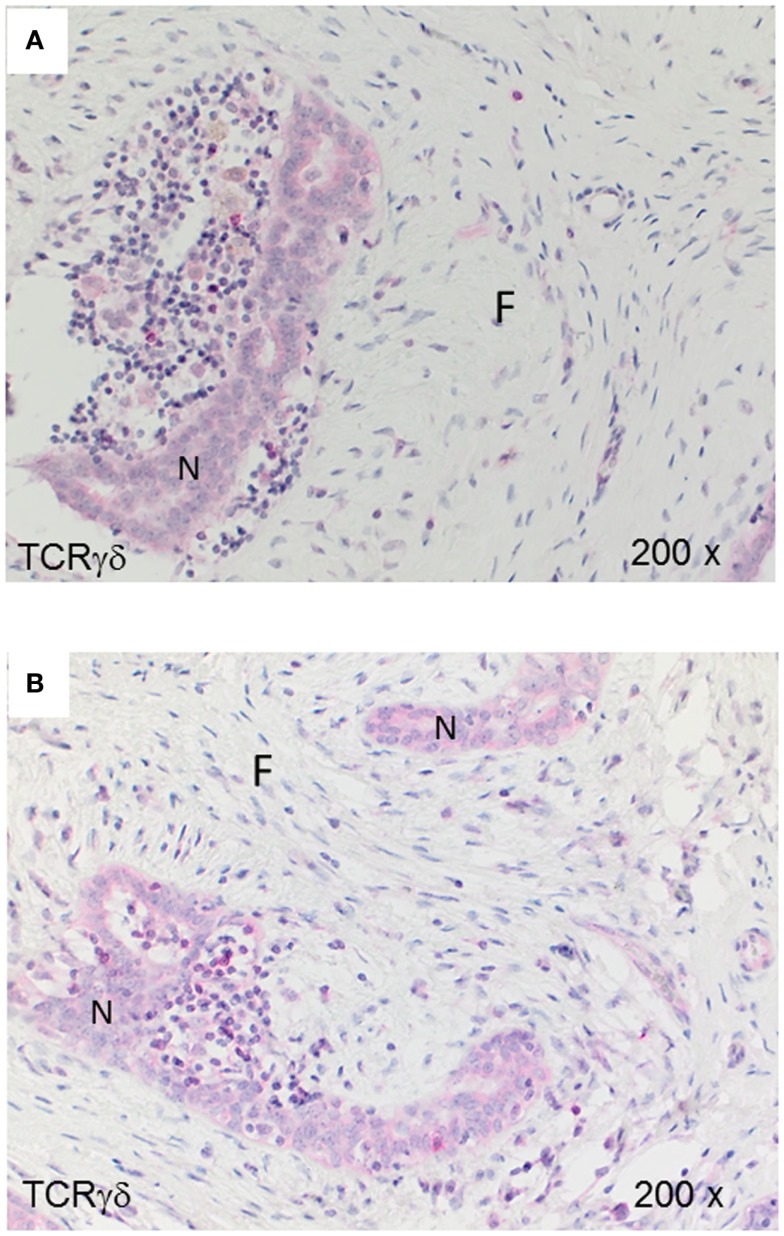
**TCRγδ^+^ T-cells in benign proliferative breast disease**. TCRγδ IHC in two representative fibroadenomas (from seven cases analyzed). Fibrotic tissue (“F”) and normal breast tissue (“N”) are marked. There is *a small* lymphocytic infiltrate with very few TCRγδ^+^ cells.

For comparison with the malignant tumors IDC and MBC, we analyzed TCRγδ^+^ cells in fibroadenomas (*n* = 7) that are benign breast lumps composed of two elements, epithelium and stroma. Some TCRγδ^+^ T-cells were present in the lymphocytic infiltrates in fibrocystic breasts (Figures 4A,B), rarely at the border or within the epithelial component, but the amount of TCRγδ^+^ cells was much lower than in the TNBC.

### Activated caspase-3^+^ tumor cells in triple-negative IDCs and MBCs

**Figure 5:**
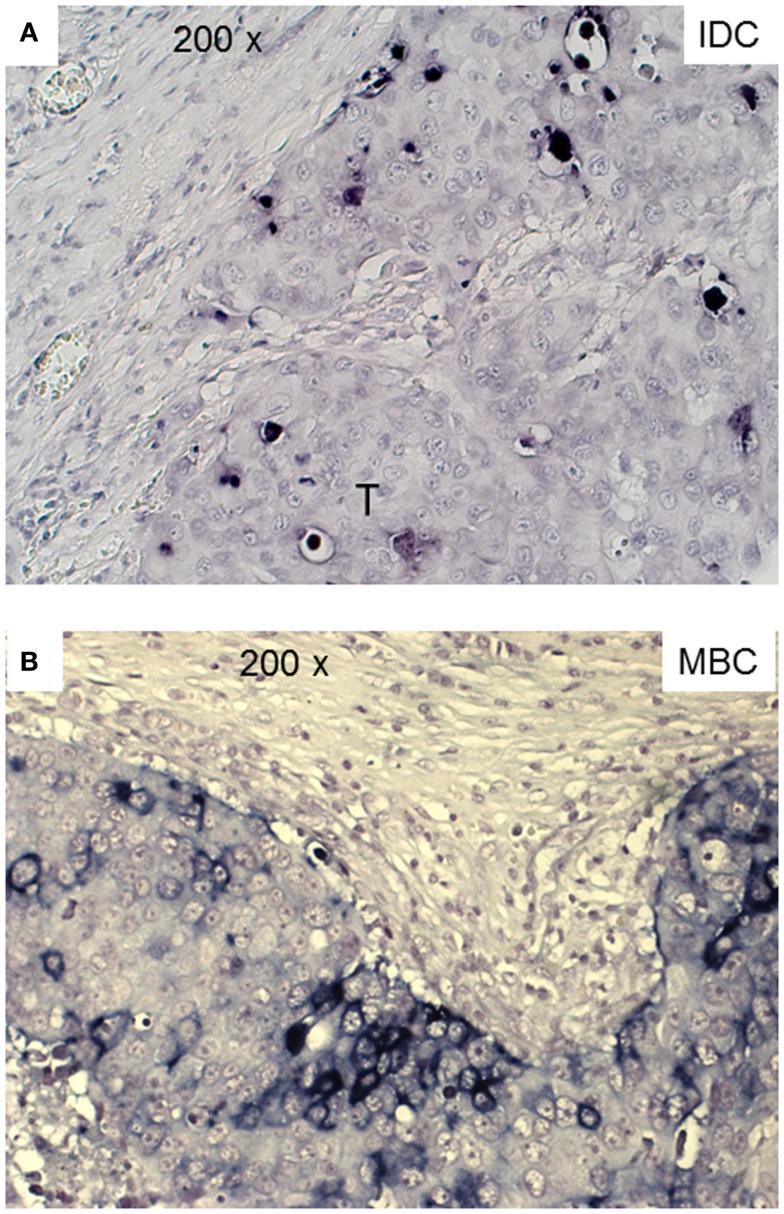
**Apoptotic tumor cells in triple negative IDC and MBC**. Cleaved-caspase-3^+^ tumor cells are detected in a representative case of IDC and MBC by a dark blue chromogen within the tumor area.

Cells positive for cleaved-caspase-3 (CC3) are undergoing apoptosis that could be induced by interaction with cytotoxic T-cells (39). We stained our FFPE tumor sections by IHC for the presence of activated caspase-3. Apoptotic tumor cells were detectable in both types of TNBC. There were significantly more CC3^+^ cells in the MBC-type than in the IDC type of TNBC (Figure 5; Table 3).

## Discussion

The functional importance of TILs in breast cancer is controversial. Most studies show that tumor-infiltrating CD8^+^ lymphocytes in breast cancer show a positive correlation with patient survival (15–18, 40). FOXP3^+^ regulatory TILs were a favorable prognostic factor in the HER2^+^/ER^−^ breast cancers, but an adverse prognostic indicator in ER^+^ breast cancer (19, 20, 41). In this study, we investigated by IHC the presence of γδ T-cells in human TNBCs comparing the IDC- and MBC-type tumors. TNBCs have attracted much attention in recent years because there are no targeted therapies for this group of breast cancers and because their profile overlaps with that of “basal-like carcinomas” (42). Histologically, TNBCs are more often the IDC type than the MBC one and are frequently displaying prominent lymphocyte infiltrates. To our knowledge, this is the first IHC analysis of γδ T-cells within TILs in TNBCs and the first study using FFPE material. Previous studies detected γδ T-cells in human breast carcinomas by IHC in frozen sections (34, 43). We found significant numbers of γδ T-cells as constituents of TILs in both the IDC- and the MBC-type of TNBC. In most IDCs, the γδ T-cells were preferentially located in the stroma and to a lesser degree in the tumor parenchyma. In MBCs, the γδ T-cells were mainly present within the tumor epithelium or at its invasive border (Figure 2; Table 3). The intratumoral infiltration by γδ T-cells in IDCs was heterogeneous. Most IDC specimens showed relatively few γδ T-cells in the tumor parenchyma in comparison with MBCs. However, in some IDCs, a manifest intratumoral γδ T-cell infiltrate was present (Figure 3). This may be related to the fact that TNBCs themselves constitute a heterogeneous subgroup, with some tumors conceivably having an intraductal and a medullary component and thus in some cases, distinguishing between the IDC- and the MBC-type may be difficult (44). What could be the reason for the differences that we observed in the γδ T-cell infiltration patterns between most IDCs and the MBCs? Potential explanations include different antigens for γδ T-cells expressed by the tumor cells or different galectins (45) or chemokines such as CCL2 (46) present in the particular tumor microenvironment. The apoptotic tumor cells as detected by CC3 expression (Figure 5) might reflect the intratumoral infiltration by cytotoxic T-cells, such as γδ T-cells that were in direct contact with the tumor cells (Figures 2 and 3). This is compatible with previous findings showing that there are more apoptotic tumor cells in MBC than in IDC (47, 48) and could be linked to the overall better prognosis of MBC.

We found that γδ T-cells are rare in normal breast tissues (Figure 1) and scarce within the lymphocytic infiltrates in fibroadenomas (Figure 4) suggesting that γδ T-cells are actively infiltrating TNBCs. The inflammatory immune responses or soluble factors secreted by the tumor cells might induce infiltration by γδ T-cells in breast carcinomas. For instance, it is possible that some TNBCs or “basal-like” breast carcinomas (49) secrete soluble chemokines attracting γδ T-cells. Also, it is conceivable that TNCBs may express γδ T-cell-recognizable antigens that are absent in other breast carcinomas and normal breast tissues. γδ T-cells in breast carcinomas could play a protective role as observed for CD8^+^ T-cells. However, one study by Ma et al. performed on frozen sections from a heterogeneous group of breast cancers suggested that intratumoral γδ T-cells correlated with the HER2 expression status, breast cancer progression and poor patient survival rates (43). These findings are compatible with the observation that breast cancer-derived γδ regulatory T-cells induce immunosenescence, resulting in suppression of innate and adaptive immunity (50). In the study by Ma et al. (43), the patients’ tumors were heterogeneous and the γδ T-cell numbers correlated with Tregs, therefore, it is not possible to exclude that other variables than γδ T-cells were involved in tumor progression.

It might be interesting to investigate the TNBC infiltrating γδ T-cells at the molecular level to define their variable gene expression and whether they can recognize breast cancer cell lines. A previous study in colon cancer suggested that intratumoral Vδ1^+^ T-cells were cytotoxic and secreted interferon-γ toward epithelial tumor cells. Our preliminary results where we isolated γδ T-cells from TNBCs by microdissection followed by single-cell PCR suggest that the γδ T-cells in these tumors do not represent the Vγ9Vδ2 population in the blood, but that they express the Vδ1, Vδ3, and Vδ4 genes (data not shown).

## Conflict of Interest Statement

The authors declare that the research was conducted in the absence of any commercial or financial relationships that could be construed as a potential conflict of interest.
